# Women’s Perceptions about Breastfeeding: A Preliminary Study

**DOI:** 10.3390/children7060061

**Published:** 2020-06-12

**Authors:** Cecilia Obeng, Stephanie Dickinson, Lilian Golzarri-Arroyo

**Affiliations:** 1 Department of Applied Health Science, School of Public Heath, Indiana University, Bloomington, IN 47405, USA; 2 Department of Epidemiology and Biostatistics, School of Public Heath, Indiana University, Bloomington, IN 47401, USA; sd3@indiana.edu (S.D.); lgolzarr@indiana.edu (L.G.-A.)

**Keywords:** breastfeeding, infants, breast milk

## Abstract

Background: Breastfeeding rates are low in many communities in the United States and require attention to come up with ideas that will help increase breastfeeding. This study investigated the effects of income, age, race and education on mothers’ perceptions about breastfeeding and whose needs and views should be considered in a women’s breastfeeding journey. Methods: A survey was distributed via email and Facebook to 525 participants; 453 participants (86.3%) responded to the survey. Results: Younger adults were more likely to agree that fathers should have some say about breastfeeding. Those earning USD 0–USD 50,000 were more likely to agree relative to those with higher incomes on children being entitled to mother’s milk, and children’s needs over-riding those of others. There was a statistically significant difference by education about women's wishes about breastfeeding being considered more important than those of their spouses. However, there was no statistically significant difference for any demographic group for breastfeeding decisions coming from women only. On women’s breasts being primarily for infant's nutrition, people who earned USD 0–USD 50,000 were more likely to agree relative to those who earned more than USD 50,000; younger adults were significantly more likely to agree. Those who earned USD 0–USD 50,000 were more likely to agree relative to those in other income brackets that extended family members should have input regarding babies being breastfed; minority participants were significantly more likely to agree relative to white participants; those with less than 4-year college education were more likely to agree relative to those with a minimum of four-year college education. Younger adults were more likely to agree that employers must provide extended maternity leave to make it easier for mothers to breastfeed. On *women having the right to breastfeed in public places,* younger adults were significantly more likely to agree compared to older adults. Conclusion: Women have favorable views about breastfeeding and prefer being in charge about decisions to breastfeed.

## 1. Introduction

The process of breastfeeding is based on habits, standards, and behaviors existing in each society [1]. On family members' role towards breastfeeding, researchers maintain that family members (especially grandmothers) and healthcare providers’ prenatal opinions are significant in women's breastfeeding decisions with family members or healthcare providers’ preference for breastfeeding leading to mothers’ decision to initiate breastfeeding [2,3]. 

Furthermore, research has demonstrated that fathers who are educated and support breastfeeding perform an important role in women’s breastfeeding journeys [4]. Indeed, fathers who are engaged and support mothers’ breastfeeding positively influences mothers' decision to breastfeed [5]. 

In a study on parental attitudes toward breastfeeding and the extent to which such attitudes are predictive of the method of infant feeding at discharge from hospital, Shaker and colleagues noted that parental attitudes toward breastfeeding are stronger predictors of infant feeding choice than sociodemographic factors [6]. As such, attitude toward breastfeeding is a significant factor, with paternal infant feeding attitude significantly predicting the choice of feeding method used by mothers [7]. 

On breastfeeding in public, research on fathers’ thoughts on breastfeeding and its implications for a theory-based intervention found that although participants value breastfeeding's healthful benefits, they are uncomfortable with women breastfeeding in public and therefore see feeding with infant formula as a more convenient mechanism as compared to traditional breastfeeding [8]. 

In a study that explored the dimension of maternal sexuality to clarify the role of fathers influence, the researchers revealed that the breastfeeding behavior of women is in direct response to both the presence of men when breastfeeding in public and partner interrupted intimacy, defined as how children’s breastfeeding behavior interrupts parents’ intimacy [9]. This, according to the authors, highlights “the dilemma of maternal sexuality as women struggle with the sexual fluidity of their breasts in social and intimate contexts” [10,11].

An observation of the above literature points to discussions about maternal sexuality as well as fathers, societies, and families’ influence on breastfeeding. What is missing in the existing literature is a single study that examines whose needs should be viewed as most important when considering breastfeeding as well as how and the extent to which the demographic variables of age, income, education, and race, influence mothers’ perception about breastfeeding. The aim of this study, therefore, is to fill the above-mentioned gap.

## 2. Materials and Methods

A questionnaire on breastfeeding was developed to survey women’s opinions on breastfeeding. The questionnaire administration was carried out between August and November of 2018 after an approval from the authors’ Institutional Review Board with an IRB approval number 1808902769. To participate in this study, a respondent had to be 18 years of age and older, and a resident of the United States. Participants were recruited via email and Facebook through snowballing; a non-probability data gathering method. It involved purposive sampling whereby we began with a small population of Facebook friends and co-workers who were known to us. We subsequently increased the number of the research participants by asking those already recruited to identify other participants to participate in the study. Using the snowballing and purposive sampling method gave us the opportunity to have an expanded sample that we could not have otherwise reached. In order to obtain authentic representative data, we also sent e-mails to participants (the Facebook friends and our co-workers) in different age groups and backgrounds. The email addresses were obtained from our Facebook friends and co-workers.

The survey instrument consisted of two main sections: respondent’s demographics and the main variables of interest. Demographic data were collected for age, income, race, and education. Respondents were asked to indicate agreement on 10 items on breastfeeding culture. With five point response options ranging from *Strongly Disagree* to *Strongly Agree* with 1 being Strongly Disagree and 5 being Strongly Agree. The survey was pilot tested for clarity by 30 respondents.

### 2.1. Data collection

Qualtrics Survey Software was used to implement the survey online, and it was distributed via email and Facebook in the research teams’ social networks. The web-based survey provided us the opportunity to obtain large numbers of respondents across the country versus traditional sampling techniques with respect to paper forms and geographical location. 

### 2.2. Data analysis

Survey responses were downloaded from Qualtrics for analysis in R software. Descriptive statistics were calculated and compared for each demographic variable. We performed overall mean Standard Deviation (SD) for each research statement/question. In the results, we have star(s) denoting the differences over all groups of each characteristic with the letters (a, b, c, d) denoting the significant differences on post-hoc pairwise analysis. The threshold for declaring statistical significance is a p-value of less than 0.05.

## 3. Results

Four hundred and fifty-three people responded to the survey. The recruitment method resulted in a sample that was 82% White and highly educated (77%) with or more years of college education. Twenty-four percent (24%) of the respondents were aged between 18–29, 48% between 30–39, 19% between 40–49 and 8% between 50–69. On income: 20% earned between USD 0–USD 50,000; 35% between 50,001–USD 95,000; and 44.3% earned USD 95,001 or more. Participants demographics and responses about perceptions on breastfeeding are shown in Table 1 and Table 2 below.

Perceptions on each of the statements/questions about breastfeeding were compared by race, education, income, and age with results presented in Table 2. 

Regarding whether *a baby’s father should have some say about breastfeeding for a baby*; there was a statistically significant difference between the age groups (*p* < 0.001). Post-hoc pairwise comparison revealed that younger adults (18–29 years) were significantly more likely to agree compared to those between 30 and 39 years of age as well as middle-aged adults (40–49 years). There was no statistically significant difference by race, income or education. 

For the statement, *“A child is entitled to mother’s milk, and her/his needs must over-ride those of others”* there was a statistically significant difference by income (*p* < 0.001), age (*p* < 0.5) and education (*p* < 0.001). Post-hoc pairwise comparison revealed that those who had an income of USD 0–USD 50,000 were more likely to agree relative to those with income USD 95,001 or more. Likewise, those with income between USD 50,001 and USD 95,000 were significantly more likely to agree relative to those with income USD 95,001 or more. Regarding age, younger adults (18–29 years) were significantly more likely to agree compared to those between 30 and 39 years of age as well as middle-aged adults (40–49 years).

With respect to women's wishes about breastfeeding being considered more important than those of their spouses or partners, there was no significant statistically difference for any demographic group except education (*p* < 0.05). There was also no significant statistically difference for any demographic group for the statement, *“Breastfeeding decisions should come from the woman only”.*

On the statement, “*women’s breasts are primarily for infant's nutrition”*, there were statistically significant differences by income (*p* < 0.01), age group (*p* < 0.001) and education (*p* < 0.001). Post-hoc pairwise comparison revealed that those who had an income of USD 0–USD 50,000 were more likely to agree relative to those with income USD 95,001 or more. Likewise, those with an income between USD 50,001 and USD 95,000 were significantly more likely to agree relative to those with income USD 95,001 or more. Relative to other age groups, younger adults (18–29 years) were significantly more likely to agree. Furthermore, relative to middle-aged adults (40–49 years), those between the ages of 30–39 years were significantly more likely to agree.

Regarding the assertion, “*Extended family members should have an input regarding the babies being breastfed by their mothers”,* there were statistically significant differences by income (*p* < 0.001), race (*p* < 0.01) and education (*p* < 0.05). Post-hoc pairwise comparison revealed that those who had an income of USD 0–USD 50,000 were more likely to agree relative to those in other income brackets. Likewise, those with income between USD 50,001 and USD 95,000 were significantly more likely to agree relative to those with income USD 95,001 or more. Regarding race, minority participants were significantly more likely to agree relative to White participants. Likewise, those with less than 4-year college education were significantly more likely to agree relative to those with a minimum of four-year college education. 

An observation of the data showed that there was no statistically significant difference for any demographic group except age (*p* < 0.001) for the statement, “*Employers must provide extended maternity leave to make it easier for mothers to breastfeed”.* Post-hoc pairwise comparison revealed that, relative to middle-aged (40–49 years) and adults (50–69 years), younger adults (18–29 years) were significantly more likely to agree. Likewise, those between the ages of 30–39 years were significantly more likely to agree relative to middle-aged adults and older adults. 

On the question of whether *women should have the right to breastfeed in public places,* there was no significant statistical difference for any demographic group except age (*p* < 0.001). Post-hoc pairwise comparison revealed that younger adults (18–29 years) were significantly more likely to agree relative to older adults. Likewise, those between the ages of 30–39 years were significantly more likely to agree relative to older adults. Furthermore, middle-aged adults were significantly more likely to agree relative to older adults. 

Finally, with respect to the statement, “*a woman can do whatever she wants with her own breasts, regardless of what other people think”,* there was a statistically significant difference by income (*p* < 0.05) and education (*p* < 0.05) but not by age or race. Post-hoc pairwise comparison revealed that respondents with an income of USD 95,001 or more were more likely to agree relative to those who earned USD 50,001–USD 95,000. Respondents with less than 4 years of College education were more likely to agree to the statement. 

## 4. Discussion

This study broadly examined mothers’ perceptions about breastfeeding. Particularly, it investigated the effects of income, age, race and education on mothers’ perceptions about breastfeeding. On the question of a baby’s father having some say about breastfeeding, our overall findings indicated neither agreement nor disagreement. Previous researchers, however, confirmed male partners’ support for and favorable attitude towards breastfeeding as being an important, and, sometimes, their being the most important factor associated with breastfeeding initiation and sustenance [10]. Furthermore, fathers’ lack of support for breastfeeding negatively impact breastfeeding whereas their support bolsters breastfeeding [11]. Indeed, research has shown that husbands’ attitudes toward breastfeeding are stronger predictors of infant feeding choice than commonly cited sociodemographic factors [12]. Furthermore, the decision of mothers who decided never to breastfeed was significantly associated with the infant's father or other extended family members’ (such as grandmothers’) preference for only formula feeding [2,6]. In our study, there was a statistically significant difference by age regarding fathers having some say about breastfeeding. Younger adults were more likely to agree that fathers should have some say about breastfeeding, but there was no effect of income, race or education. What our findings and those identified in the literature suggest is the important fact about young mothers welcoming the input of their spouses in breastfeeding. Thus, attempts at scaling up breastfeeding, besides targeting mothers, must also target fathers and encourage them to show interest in, and encouraging their spouses to breastfeed their babies. Attempts should also be made to garner interest among older women and women of various income levels, race, and education to impress upon them to see the benefit of fathers’ role in infants’ breastfeeding.

With regards to the statement, “*baby's extended family should ensure that the baby's mother is breastfeeding”*, our findings, on the whole, indicated neither agreement nor disagreement across the board. However, there was a statistically significant difference by income, race and education. This finding is significant given that there are many studies that point to input of spouses, other family members and friends as impacting breastfeeding initiation and sustenance [6,7,12,13]. Indeed, research in Australia showed that mothers chose to breastfeed because of family influences, advice from others, among others [14]. Given the relevance of education, income and race of family members in influencing nursing mothers’ decision to breastfeed, it is important for perinatal educators to provide relevant healthful education to mothers and family members about the benefits of breastfeeding. Specifically, fathers and extended family members must be educated about breastfeeding and subsequently be encouraged to become active learners about the breastfeeding process and its benefits to infants and mothers as well as strategies that support breastfeeding.

On the statement, “*children are entitled to mother’s milk*, and the *children’s needs over-riding those of others”* our overall findings did not indicate agreement nor disagreement among all the participants. An observation of past research supports the assertion regarding children’s entitlements to breast milk. Specifically, research has shown that infants have the right to be free from hunger, have safe access to breast milk, and must therefore have adequate food [15]. However, in the United States, even though high income and well-educated mothers are far more likely to breastfeed their infants to reach the six-months target, babies born to low income, less-privileged, and less educated mothers do not reach the six-month breastfeding target. In particular, whereas 92% of college educated mothers ever breastfed their children, only 69.3% of high school mothers breastfed their infants [15,16,17]. Moreover, well-off parents saw breastfeeding as desirable and breastfed their infants for 6 months and over; the reason for seeing breastfeeding as desirable and for breastfeeding for six months was that they had infrastructure that supported breastfeeding and worked in jobs that provided what was needed to breastfeed. White women also breastfed their infants more than non- whites. On the contrary, non-white women from households with the lowest incomes with new mothers working in non-breastfeeding friendly office environments experienced lower rates of breastfeeding [17]. Note, however, that in our study respondents earning USD 0–USD 50,000 were more likely to agree (to the statements “*children are entitled to mother’s milk”*, and the “*children’s needs over-riding those of others”*) relative to those with higher incomes. We showed further that those with less than 4 years of college education were more likely to agree than those with 4 years of college education or higher. What our findings suggest is that a lot more education is needed to improve the perception of mothers in the high income levels, women older than thirty and women with 4 years of college education and over with regards to children’s entitlement to mothers’ breast milk.

On the question of whether *women should have the right to breastfeed in public places*, there was no statistically significant difference for any demographic group (income, education and race) except age. Our findings support those by researchers who demonstrated a fear of breastfeeding in public in young women, low income women, and immigrant women in Western countries [13,18]. However, our findings differ from researchers who discovered that Western societies’ inability to divorce the biologically functional and sexual role of women’s breasts results in breastfeeding being seen as a private function and consequently breastfeeding in public being perceived as inappropriate [13]. Furthermore, our findings differ from those who discovered that male partners and members of the public were uncomfortable with women breastfeeding in public [8,9]. Indeed, some of the research cited above even saw formula feeding to be more convenient than breastfeeding [8], whereas breastfeeding mothers struggled with the sexual fluidity of their breasts in social and intimate contexts [9]. Other researchers have shown how negative and even aggressive attitudes and actions by the public have caused mothers not to breastfeed in public. One study noted that many mothers have been asked to stop breastfeeding or to leave when attempting to breastfeed in public places while other breastfeeding women are publicly shamed and thus embarrassed by others thereby forcing them to choose to feed their infants on supplementary formula or completely give up breastfeeding [18,19]. 

What is important, though, is that in the United States many states have adopted laws asserting the right to breastfeed in public view, whereby ‘public’ includes places frequented by the general public (privately or publicly owned), restaurants, shopping malls, stores, and sports stadiums. In our study, younger, adults (18–29 years) were significantly more likely to agree relative to older adults that women should have the right to breastfeed in public places. The clinical implications of our study taking cognizance of our findings and of the above cited studies suggest that despite objections that may be raised by some regarding public breastfeeding, it is concomitant for mothers and the general public to consider the nutritional needs of infants as superseding any such objections. States must also do more to protect women from possible harassments to help ensure that infants are properly nourished when needed and to avoid infant hunger and malnourishment [20,21]. 

The above discussion has relevance for the question regarding whether *a woman's breasts are primarily for infant's nutrition*. With regards to this question, there were statistically significant differences by income, age, and education but not by race. Our findings are different from those in the literature. Indeed, research has shown that the sexualization of the breast in the western world created a situation where breasts were viewed as not primarily for infant feeding. Findings from our study, however, suggest that respondents with an income of USD 0–USD 50,000 were more likely to agree relative to those in the other income groups; also, adults between the ages of 30–39 were significantly more likely to agree than over forty years of age. Furthermore, those with less than 4-year college education were more likely to agree than those with a longer-than-4-year college degree; there was, however, no effect of race. The clinical implications of the difference regarding age, education and income as well as what obtains in the literature are as follows: attempts aimed at bolstering breastfeeding, and especially at influencing perceptions of the breast, must emphasize the nutritional, and more especially, the health benefits of breast milk. Such attempts ought to deemphasize sexualization of the breast. These attempts must target women of all ages, income brackets, and educational levels.

On the statement, “*a woman should be able do whatever she wants with her own breasts, regardless of what other people think”*, our overall finding indicated neither agreement nor disagreement. There was a statistically significant difference by income and education but not by age or race. Research in this area is found in those that sexualize the breast, explore whether a father must have some say about breastfeeding, investigate whether a woman's breasts are primarily for infant's nutrition, among others. A systematic observation of the literature in the above mentioned areas points to restrictions, both implicit and explicit about what women can and cannot do with their breasts. Restrictions on the breast are based on stereotypes about women’s breasts, religious beliefs, and male preferences [22]. Research has shown that middle age women who go topless in some parts of the western world are either cited or harassed making it impossible for them to do whatever they want with their breasts [23]. From the above literature, we learn about women not being able to do whatever they want with their breasts. As noted earlier, women’s inability to bare their breasts in public impact their ability to breastfeed in public. In our study, on the statement, “*a woman should be able do whatever she wants with her own breasts, regardless of what other people think”,* respondents with an income of USD 95,001 or more were more likely to agree relative to those who earned USD 50,001–USD 95,000 or less. Furthermore, respondents with less than 4 years of college education were more likely to agree to the statement. The above-cited literature and the results of our study have implications for clinical practice. In particular, the absence of freedom to do whatever they want with their breasts presupposes coercion that women are subjected to which in turn negatively impact their ability to freely nurse their infants, especially in public. Viewed within the context of our study, it could be argued that clinicians, public health experts, social workers, and other stakeholders, ought to pay attention to problems or issues faced by women of the various demographics identified in this study with the view to working with them to mitigate the problems they face in breastfeeding their babies in various social and sociological ecologies.

On the issue of whether *a child’s needs and entitlement to mother’s milk ought to override those of others*, we discovered a statistically significant difference by income, age and education but not in race. Our study supports those by researchers who showed behavioral perceptions and attitudes (positive and negative) about breastfeeding impacting breastfeeding initiation and sustenance [9,13]. Their findings also showed that people with low to lower middle incomes were more likely to agree with a child’s needs and entitlement to mother’s milk overriding those of others. Furthermore, post-hoc pairwise comparison in our study revealed that low income women were significantly more likely to agree (to the statement that a child is entitled to mother’s milk, and that her/his needs must over-ride those of others) relative to those in higher income groups. Our study supports a study that saw low income Latina women as favoring breastfeeding [24,25]. Despite the fact that low-income respondents and younger adults and women with less than 4 years of education were more likely to agree with the assertion that children’s needs and entitlement to mother’s milk ought to override those of others, research has shown that 78% of infants from low income families in the United States received formula supplementation in the hospital even where there was no clear medical need for infants receiving such supplementation [26,27]. 

Furthermore, research has shown that younger women, particularly <20 yrs, less educated women, and women with low-income and socioeconomic status are less likely to breastfeed and would benefit from increased support [28]. The contrast of our study findings with those of the above-cited literature points to the need for greater support and education for mothers to scale up breastfeeding, more especially because the above-cited studies all acknowledge the nutritional and healthful significance of breastmilk in boosting the immune system of infants and in helping to protect infants from childhood killer diseases that are easily mitigated by well-organized breastfeeding schedules [29].

Another important observation of our study was the fact that there was no significant statistically significant difference regarding whether *breastfeeding decisions should come from the women only*. Research has shown that in the United States women who choose breastfeeding tend to be more often married, White or Hispanic, 30 years or older, college educated, and living in the Pacific Northwest or New England [30]. However, a study conducted in Canada showed that mothers’ income and social status did not affect the woman’s decision to breastfeed [31]. Another study, using both race and socioeconomic status, however, reported that differences were more prominent related to socioeconomic status than race. They also reported that a mother’s intention to breastfeed was the single most significant factor predictive of her breastfeeding behavior [32]. Even though the above studies did not specifically seek to find out whether breastfeeding decisions should come from the women only, they amplify the views of women who choose to breastfeed suggesting absence of coercion and hence freedom in the choice process. The above-cited literature and our own findings, which are around 3.3 on a scale of 1–5, point to the relevance income, education, age and education in choices made by mothers to breastfeed. It also points to the fact that given the important role by mothers in determining whether or not to breastfeed and in view of the fact that maternal age, income, race, and educational levels all play a role in breastfeeding initiation and duration, it is incumbent upon perinatal and public health personnel as well as other stakeholders to educate mothers about the health and nutritional significance of breastfeeding and the need to initiate and manage it at the appropriate time.

Furthermore, given the preponderance of literature on the sexualization of breasts in Western societies [8,9], and the fact that men’s and other family members’ attitude and lack of support can negatively impact successful breastfeeding [12,13], it is important to let women’s views be the leading source of breastfeeding initiation and sustenance. Indeed, just cited suggestion is supported by one of our study findings regarding the question of *whether women’s wishes about breastfeeding was more important than those of their spouses* where we discovered a statistically significant difference by age. Our study bolsters those that showed that maternal, not paternal, infant feeding attitude more significantly predicted the choice of feeding method used by mothers in the antenatal period [7]. Such studies also noted how expectant mothers were sensitive to the way and manner in which healthcare professionals supported and informed mothers about infant feeding choices [7].

Finally, for the statement “*employers should provide extended maternity leave to make it easier for mothers to breastfeed”*, most of our respondents strongly agreed to the statement. There was a statistically significant difference by age (*p* < 0.001) but not by income, race or education. Research has shown that among other factors, a higher education level is a significant predictor of continued breastfeeding for more than 6 months after returning to work [32]. Particularly, the above study showed that age (young age), mother’s education level (lower education), insufficient breastfeeding knowledge, among other factors, were all associated with discontinuing breastfeeding after returning to work [33]. Moreover, research has indicated that extended maternity leave benefits low income mothers because short maternity leave requires them to purchase breast pumps that cost over USD 380, which is not affordable to such mothers [34]. Furthermore, the Family Medical Leave Act (FMLA) permits eligible employees to take a total of 12 workweeks of leave during any 12-month period for their child. Moreover, research has shown that longer maternity leave is associated with longer duration of breastfeeding [35] and that income (especially, paid leave) is more likely to influence the decision by mothers to breastfeed and how long to breastfeed. Specifically, low-income mothers are more likely to stay home and breastfeed for longer periods of time if they are offered paid maternity leave [36].

In our study, we indicated that post-hoc pairwise comparison revealed that younger adults (18–29 years) were significantly more likely to agree to the statement (“*employers should provide extended maternity leave to make it easier for mothers to breastfeed”*) relative to those aged between 30–39, middle-aged adults, and older adults. What the above-cited studies and our findings suggest is that age, income, and education are associated with mothers quest for employers to grant extended maternity leave to enable them breastfeed for longer periods of time. Thus, to encourage longer periods of breastfeeding, employers must provide extended maternity leave and create conducive work environment that help to promote breastfeeding among the young adult mothers in particular, and nursing mothers of all ages, income, racial and educational backgrounds, in general.

### Limitations

A limitation of this study is the recruitment method, which involved surveying a convenience sample of individuals from the authors’ social networks, who are likely to be from similar backgrounds and to share beliefs about the topic under investigation. Future studies could look at samples involving equal numbers of people from the different demographics used in this study. Furthermore, this study took place via Facebook and Email. This suggests that some people without Facebook and email access who could not meet face-to-face with any of the research team were eliminated from the study. Despite these limitations, the study findings uncovered important facets about women’s innermost perceptions about the broad topic of breastfeeding and the relevance of such variables as age, race, education and income on women’s breastfeeding journey; something which no other study has explicitly done. Detailed benefits of this study are discussed in the next section. 

## 5. Conclusions

This study determined that age, income, race and education influence mothers’ perceptions about breastfeeding, including where to breastfeed. Thus, even though women viewed themselves as the main persons in their breastfeeding ecology, the level of income and education as well as their various age groups shape their perceptions about who should be involved in breastfeeding decision-making. We learned, for example, that those with less than 4 years of college education were significantly more likely to agree, relative to those with a minimum of 4 years of college education, that extended family members must have a say in decisions about breastfeeding. Moreover, the sexualization of the breast has made breasts as being for men and sex and hence made breast-feeding infants abnormal and destabilizing. This, we noted, has negatively affected women’s ability to breastfeed in public [8,9].

Furthermore, this study has shown the relevance of race in determining whether extended family members’ input is needed in breastfeeding initiation and sustenance, where to breastfeed, and whether women’s breasts are primarily for breastfeeding. Thus, from our study and some of the above-cited literature/research, we discovered, for example, that non-whites (minority participants) were more likely to agree relative to white participants on extended family’s input being needed or necessary regarding the decision to breastfeed.

These and the other findings are very important, because efforts to get mothers to breastfeed must be geared primarily at women in general and mothers in particular. However, this is not to suggest that fathers and the other family members and the community have no role in breastfeeding intervention and promotion; as we learned from the results and discussion, fathers, family members and the community have a role to play but these should be situated within the limits set by the mothers. Thus, any attempt to force mothers not to breastfeed or to breastfeed against their will could be catastrophic, since this study shows women recognize themselves as the mainstay or basis in their breastfeeding journey. Additionally, this also implies that mothers’ views must be respected and be made part of any intervention strategies or models to ensure optimal health results for infants. Indeed, it could be argued that one of the most important contributions of this study to the field of Public Health relates to women’s rights regarding their breastfeeding journey. Thus, based on the results of our study, although mothers respect and may sometimes even seek the opinions and perspectives of their spouses as well as those of other family and their communities on breastfeeding, they should be the main and final decision makers in breastfeeding initiation and sustenance as well as where to breastfeed.

Future research will target culturally congruent strategies that will be feasible and effective in different communities in the United States. In addition, interventions that attempt to encourage mothers not to close their eyes/ears to effective and appropriate suggestions from others in the breastfeeding ecology must be conscientiously promoted to help improve, increase and sustain breastfeeding. Such interventions must take cognizance of mothers’ views, their comfort, and the welfare of the breastfeeding child, to avoid miscommunication and failure.

## Figures and Tables

**Table 1:** Participants Demographic.

Participant Information	453
**Income (regrouped)**	
USD 0–USD 50,000	90
USD 50,001–USD 95,000	156
USD 95,001 or more	201
**Age**	
18–29	113
30–39	217
40–49	87
50–69	35
**Race or ethnicity**	
Minority	77
White	371
**Education**	
Less than 4-year	112
4-year or higher	341

**Table 2:** Breastfeeding perceptions by demographic groups (1 = Strongly disagree to 5 = Strongly agree).

	Overall Mean	Income			Age Group				Race		Education	
Research Questions/Statements	Female	USD 0–USD 50,000	USD 50,001–USD 95,000	USD 95,001 or more	18–29	30–39	40–49	50–69	Minority	White	Less than 4-year	4-year or higher
	*N* = 453	*N* = 90	*N* = 156	*N* = 201	*N* = 113	*N* = 217	*N* = 87	*N* = 35	*N* = 77	*N* = 371	*N* = 112	*N* = 341
A baby's father should have some say about breastfeeding for a baby	2.86 (1.17)	2.86 (1.16)	2.97 (1.16)	2.78 (1.16)	3.22 (1.14)^ab^	2.78 (1.13)^a^	2.54 (1.17)^b^	2.94 (1.19)***	2.92 (1.27)	2.84 (1.14)	2.99 (1.17)	2.82 (1.16)
A child is entitled to breast milk, and her or his needs must over-ride those of others	3.45 (1.28)	3.85 (1.19)^a^	3.61 (1.26)^b^	3.17 (1.26)^ab***^	3.77 (1.22)^ab^	3.33 (1.31)^a^	3.34 (1.22)^b^	3.46 (1.24)*	3.57 (1.29)	3.44 (1.27)	3.92 (1.09)	3.30 (1.30)***
A woman should consider her family's input about breastfeeding	2.24 (1.04)	2.17 (1.03)	2.22 (1.02)	2.28 (1.05)	2.27 (1.04)	2.19 (1.04)	2.29 (1.00)	2.31 (1.13)	2.38 (1.16)	2.20 (1.01)	2.04 (1.01)	2.31 (1.04)*
Breastfeeding decisions should come from the woman only	3.30 (1.17)	3.34 (1.15)	3.23 (1.16)	3.35 (1.18)	3.18 (1.20)	3.29 (1.18)	3.47 (1.11)	3.31 (1.13)	3.36 (1.26)	3.29 (1.15)	3.22 (1.18)	3.32 (1.16)
A woman's wishes about breastfeeding are more important than her spouse or partner's	3.98 (0.95)	3.86 (1.15)^a^	3.88 (0.91)^b^	4.12 (0.86)^ab*^	3.81 (1.01)	4.04 (0.92)	4.00 (0.96)	4.14 (0.91)	3.91 (1.18)	4.00 (0.90)	3.73 (1.07)	4.06 (0.90)**
A woman's breasts are primarily for infant's nutrition	3.50 (1.27)	3.74 (1.18)^a^	3.63 (1.21)^b^	3.30 (1.34)^ab**^	3.91 (1.11)^abc^	3.50 (1.27)^ad^	3.10 (1.31)^bd^	3.17 (1.34)***	3.54 (1.33)	3.49 (1.26)	3.88 (1.13)	3.38 (1.30)***
A baby's extended family should ensure that the baby's mother is breastfeeding	2.08 (1.01)	2.43 (1.14)^ab^	2.15 (1.02)^ac^	1.88 (0.89)^bc***^	2.22 (0.94)	2.07 (1.02)	1.90 (1.01)	2.14 (1.12)	2.40 (1.12)	2.01 (0.98)**	2.26 (1.00)	2.02 (1.01)*
Employers should provide extended maternity leave to make it easier for mothers to breastfeed	4.40 (0.85)	4.41 (0.79)	4.44 (0.88)	4.39 (0.82)	4.43 (0.75)^ab^	4.58 (0.74)^cd^	4.07 (1.04)^ac^	4.06 (0.97)^bd***^	4.40 (0.82)	4.40 (0.86)	4.43 (0.77)	4.40 (0.88)
Women should have the right to breastfeed in public places	4.73 (0.60)	4.74 (0.53)	4.70 (0.67)	4.78 (0.49)	4.70 (0.64)^a^	4.80 (0.49)^b^	4.75 (0.59)^c^	4.34 (0.87)^abc***^	4.70 (0.65)	4.74 (0.59)	4.72 (0.66)	4.73 (0.58)
A woman can do whatever she wants with her own breasts, regardless of what other people think	4.22 (1.07)	4.22 (1.11)	4.06 (1.15)^a^	4.35 (0.97)^a*^	4.29 (0.98)	4.24 (1.07)	4.21 (1.17)	3.91 (1.11)	4.37 (1.04)	4.20 (1.07)	4.43 (0.87)	4.15 (1.12)*

Data presented as Mean (SD), * *p* < 0.05, ** *p* < 0.01, *** *p* < 0.001; ^a, b, c, d^ same letter denotes significant differences on post-hoc pairwise comparison.
